# Association Between Metabolic Obesity Phenotypes and Alcohol-Associated Fatty Liver Disease Among US Adults: A NHANES 2013–2018 Analysis

**DOI:** 10.3390/jcm15145726

**Published:** 2026-07-22

**Authors:** Priyanka Dadha, Fatima Sayyed, Shakeel Ahmed, Alok Dwivedi, Jennifer Molokwu

**Affiliations:** 1 Molecular and Translational Medicine, Paul L. Foster School of Medicine, Texas Tech University Health Sciences Center El Paso, El Paso, TX 79905, USA; shakeel.ahmed@ttuhsc.edu; 2 Paul L. Foster School of Medicine, Texas Tech University Health Sciences Center El Paso, El Paso, TX 79905, USA; fsayyed@ttuhsc.edu; 3 Department of Biomedical Informatics, Biostatistics and Medical Epidemiology, School of Medicine, University of Missouri, Columbia, MO 65212, USA; alok.dwivedi@health.missouri.edu; 4 Department of Family and Community Medicine, Paul L. Foster School of Medicine, Texas Tech University Health Sciences Center El Paso, El Paso, TX 79905, USA; jennifer.molokwu@ttuhsc.edu

**Keywords:** alcohol-related fatty liver disease, metabolic obesity phenotypes, obesity, metabolic syndrome, NHANES, epidemiology, Hispanic population

## Abstract

**Background**: Alcohol-associated fatty liver disease (AFLD) is a major public health concern and remains one of the leading causes of chronic liver disease in the United States. Understanding the interplay between obesity, metabolic dysfunction, and lifestyle factors may help identify populations at increased risk for AFLD. **Methods**: This cross-sectional study utilized data from the National Health and Nutrition Examination Survey (NHANES) collected between 2013–2018. AFLD was operationally defined using elevated liver enzyme levels combined with risky alcohol consumption. Metabolic obesity phenotypes (MOPs) were characterized by combining body mass index categories with either metabolic health status or metabolic syndrome (MS). **Results**: The weighted prevalence of AFLD was 14% (95%CI: 12–16%). AFLD prevalence was significantly higher among males than females (17% vs. 10% respectively, *p* < 0.0001), and among Hispanics compared to Non-Hispanic Black participants (20% vs. 9% respectively, *p* < 0.0001). Compared with metabolically healthy normal-weight individuals, obese participants with MS demonstrated the strongest association overall AFLD association (adjusted PR: 6.73; 95% CI: 4.51–10.05). Furthermore, obese males with MS showed the highest odds of AFLD under both MOP schemes (MOP 1: OR: 8.19; 95% CI: 4.41–15.23; MOP 2: OR: 10.92; 95% CI: 6.86–17.37). **Conclusions**: Individuals with obesity and metabolic dysfunction demonstrated significantly higher AFLD prevalence compared with metabolically healthy normal-weight individuals. MOPs incorporating MS demonstrated stronger associations with AFLD than classifications based on metabolic health status alone. These findings support the importance of screening strategies that address both obesity and metabolic dysfunction in addition to alcohol use to better identify high-risk populations.

## 1. Introduction

Alcohol-associated fatty liver disease (AFLD) is an emerging public health concern, characterized by excessive hepatic fat accumulation resulting from chronic alcohol consumption. AFLD represents the earliest stage of alcohol-associated liver disease (ALD), a disease continuum characterized by hepatic steatosis, inflammation, fibrosis, cirrhosis, and hepatocellular carcinoma. The hepatic metabolism of alcohol generates toxic metabolites that impair lipid metabolism and damage hepatocytes, leading to hepatic steatosis and inflammation. Persistent inflammation may subsequently progress to fibrosis, cirrhosis, and hepatocellular carcinoma. Globally, approximately 5% of individuals are affected by AFLD, making it a significant worldwide health concern [1,2]. In the United States, AFLD remains one of the leading causes of chronic liver disease [3].

Recently, the conceptual framework and nomenclature of fatty liver diseases have evolved toward the broader category of steatotic liver disease (SLD), including metabolic dysfunction-associated steatotic liver disease (MASLD) and metabolic dysfunction-associated alcohol-related liver disease (MetALD) [4]. Despite these evolving definitions, ALFD terminology remains commonly used in epidemiologic studies examining alcohol-related hepatic injury. Emerging evidence suggests that metabolic dysfunction contributes substantially to alcohol-related liver injury beyond alcohol consumption alone [5].

Historically, AFLD was attributed primarily to excessive alcohol intake. However, emerging evidence demonstrates that obesity and metabolic dysfunction play important roles in disease susceptibility and progression [6,7,8]. Metabolic syndrome (MS), characterized by central obesity, hypertension, hyperglycemia, hypertriglyceridemia, and reduced HDL cholesterol is strongly associated with hepatic steatosis and alcohol-related liver injury [9]. Obesity contributes to insulin resistance, oxidative stress, and systemic inflammation, all of which may exacerbate alcohol-induced hepatocellular injury [10].

Studies integrating body mass index (BMI) and metabolic health have proposed metabolic obesity phenotype (MOP) frameworks to better characterize heterogeneity in cardiometabolic risk profiles [11,12,13]. These phenotypes (MOPs) classify individuals according to BMI category and metabolic health status or MS presence. Emerging evidence from SLD research suggests that metabolic dysfunction may be more strongly associated with disease severity than body size alone [14].

Recent evidence further supports the importance of integrating metabolic dysfunction into alcohol-related liver disease assessment. A recent study demonstrated that MASLD, MetALD, and ALD were independently associated with elevated sudden cardiac arrest risk, with risk highest among individuals with ALD [15]. Furthermore, MetALD demonstrated a disproportionate impact among younger adults [15]. Additionally, the fatty liver index (FLI), a widely used surrogate marker for SLD, has demonstrated predictive utility for cardiovascular disease and mortality across varying levels of alcohol consumption [16].

The prevalence and severity of AFLD are additionally influenced by demographic and behavioral factors that vary across populations [17]. Hispanic populations, particularly Hispanic men, experience disproportionately elevated burdens of metabolic disease and alcohol associated liver injury [18,19,20,21]. Despite the clinical importance of AFLD, relatively few studies have examined the association between MOPs and AFLD across diverse demographic groups. Therefore, this study aimed to characterize the prevalence of AFLD and examine its association with MOPs, while accounting for demographic and lifestyle factors.

## 2. Methods

### 2.1. Study Population and Data Source

**Figure 1:**
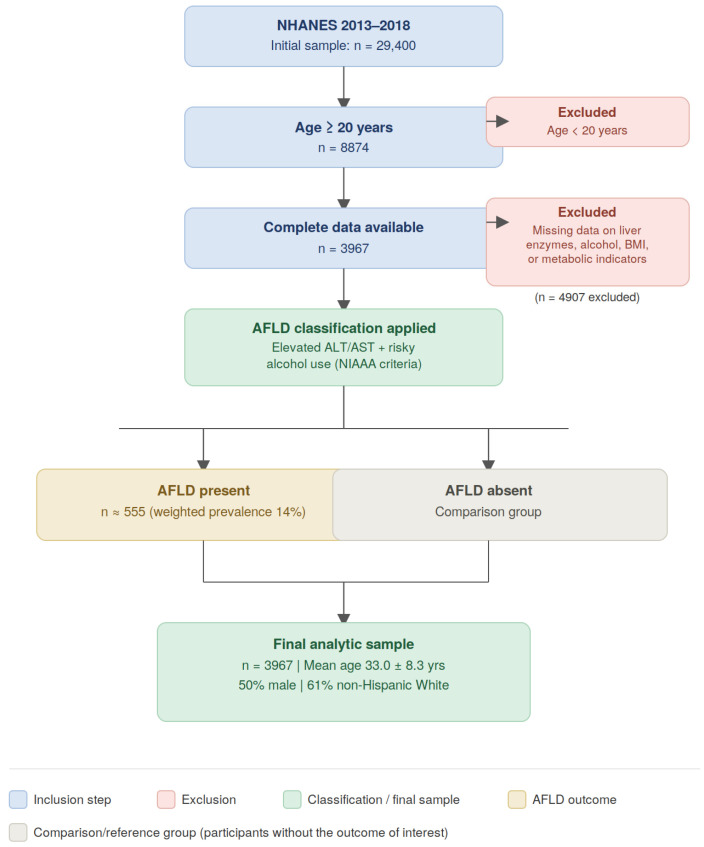
Participant selection diagram.

This cross-sectional study utilized data from the National Health and Nutrition Examination Survey (NHANES) collected between 2013 and 2018. NHANES is conducted by the National Center for Health Statistics (NCHS), using a complex, multistage probability sampling design to generate nationally representative estimates of the civilian noninstitutionalized US population [22]. Data were collected through standardized interviews, physical examinations, and laboratory analyses. Among 29,400 NHANES participants surveyed between 2013 and 2018, 8874 adults aged 18–50 had complete alcohol-use data. Participants missing liver enzyme measurements, alcohol consumption variables, anthropometric measures, MS components, or covariates were excluded. The final analytic sample included 3967 participants. A participant selection flow diagram is provided in Figure 1.

Missing data was handled using complete case analysis. Participants with missing data on liver enzyme measurements, alcohol consumption variables, anthropometric measurements, MS, or key covariates were excluded from the analysis. Although this approach preserved consistency across analyses, it may have introduced selection bias if excluded participants differed systematically from included participants.

### 2.2. Outcome Definition: ALFD

AFLD status was operationally defined using biochemical and behavioral criteria. Serum alanine- aminotransferase (ALT) and aspartate aminotransferase (AST) levels were measured using standardized NHANES laboratory procedures [23]. Elevated liver enzymes were defined using sex-specific cut-offs commonly used epidemiologic studies: ALT > 40 U/L and AST > 37 U/L for men; ALT > 31 U/L or AST > 31 U/L for women [24].

Alcohol consumption was assessed using NHANES alcohol use questionnaires. Risky alcohol use was defined according to National Institute on Alcohol Abuse and Alcoholism (NIAAA) criteria as >14 drinks per week or >4 drinks/day for men, and >7 drinks per week or >3 drinks/day for women [25]. Participants meeting both risky alcohol consumption and elevated liver enzyme criteria were classified as having AFLD.

To ensure the validity of fasting-dependent MS variables, fasting duration prior to blood draw was assessed using the NHANES variable PHAFSTHR. Participants included in analyses requiring fasting glucose and triglyceride measurements were restricted to those fasting ≥8 h prior to specimen collection, consistent with ATP III MS assessment recommendations.

### 2.3. Exposures: MOPs

BMI was calculated as weight in kilograms divided by height in meters squared (kg/m^2^) using standardized NHANES anthropometric measurements [26]. Participants were categorized as normal weight (BMI 18.5–24.9 kg/m^2^), overweight (BMI 25.0–29.9 kg/m^2^), or obese (BMI ≥ 30.0 kg/m^2^) [13].

Two MOP classification schemes were constructed by integrating BMI category with metabolic health status, consistent with established MOP frameworks in the literature [13].

MOP 1: combined BMI category with metabolic health status: Normal Weight + Metabolically healthy (NW + MH), Normal Weight + Metabolically Unhealthy (NW + MU), Overweight + Metabolically Healthy (OW + MH), Overweight + Metabolically Unhealthy (OW + MU), Obese + Metabolically Healthy (OB + MH), and Obese + Metabolically Unhealthy (OB + MU).

MOP 2: combined BMI category with MS status: Normal Weight + No MS (NW + No MS), Normal Weight + MS (NW + MS), Overweight + No MS (OW + No MS), Overweight + MS (OW + MS), Obese + No MS (OB + No MS), and Obese + MS (OB + MS).

Metabolically healthy was defined as failing to meet the threshold for metabolic unhealthiness (i.e., having fewer than two of the six Wildman criteria) [9]. MS was defined according to Adult Treatment Panel III (ATP III) criteria requiring ≥ 3 of the following: waist circumference > 102 cm in men or >88 cm in women; triglycerides ≥ 150 mg/dL; HDL cholesterol < 40 mg/dL in men or <50 mg/dL in women; blood pressure ≥ 130/85 mmHg or antihypertensive medication use; fasting glucose ≥ 100 mg/dL or antidiabetic medication use [10]. Unhealthy behavior was defined as current smoking, insufficient physical activity, or risky alcohol consumption according to NIAAA guidelines. Cardiac syndrome was defined as a self-reported history of coronary heart disease, heart attack, stroke, heart failure, or angina.

NHANES prescription medication files were additionally examined to identify antihypertensive, antidiabetic, and lipid-lowering medication use. However, because NHANES medication files do not consistently specify medication indication, some degree of misclassification may remain.

### 2.4. Current Nomenclature

To align our study with the current international consensus nomenclature, individuals meeting the criteria for AFLD were stratified into exclusive phenotypic sub-groups based on their metabolic health [4]. Participants were stratified into four severity tiers based on the number of concurrent metabolic risk factors present: pure ALD (0 factors), mild MetALD (1 factor), moderate MetALD (2 factors), and severe MetALD (≥3 factors).

### 2.5. Statistical Analysis

All analyses followed NHANES analytic guidelines incorporating survey weights, strata, and primary sampling units (PSUs) to generate nationally representative estimates [27].

Descriptive statistics summarized participant characteristics by AFLD status. Differences between groups were assessed using survey-weighted chi-square tests for categorical variables and survey-weighted *t*-tests or ANOVA for continuous variables.

Survey-weighted multivariable logistic regression models were fitted to evaluate association between AFLD and MOPs. Because the weighted prevalence of AFLD exceeded 10%, prevalence ratios (PRs) and corresponding 95% confidence intervals (CIs) were estimated using marginal standardization (predicted marginals) derived from the fitted logistic regression models rather than directly interpreting odds ratios, which may overestimate associations in cross-sectional studies with common outcomes. Adjusted models controlled for age, sex, race/ethnicity, education, smoking status, and physical activity.

All analyses were conducted using Stata version 17.0 (StataCorp LLC, College Station, TX, USA). Statistical significance was defined as a two-sided *p*-value < 0.05.

### 2.6. Ethical Considerations

NHANES protocols were approved by the NCHS Research Ethics Review Board, and written informed consent was obtained from all participants. The present secondary analysis utilized publicly available de-identified data and was exempt from additional institutional review board approval.

## 3. Results

A total of 3967 participants were included in the analysis (mean age 33.0 ± 8.3 years; 50% male). Most participants were non-Hispanic White (61%), followed by Hispanic (20%) and non-Hispanic Black (10%). Approximately one-quarter of participants had MS, and nearly one in four were classified as obese (Table 1 and Table 2).

The weighted prevalence of AFLD was 14% (95% CI: 12–16%). Prevalence was significantly higher among males compared with females (17% vs. 10%, *p* < 0.0001) and among Hispanic participants compared with non-Hispanic Black participants (20% vs. 9%, *p* < 0.0001) (Figure 1). AFLD was also higher among individuals with lower educational attainment, unhealthy lifestyle, obesity and MS (*p* < 0.0001) (Table 3). AFLD prevalence increased progressively across worsening MOPs. In MOP 1, prevalence ranged from 5% among metabolically healthy normal weight participants to 33% among metabolically unhealthy obese participants. In MOP 2, prevalence was lowest among normal-weight individuals without MS and highest among obese individuals with MS (Table 3).

**Figure 2:**
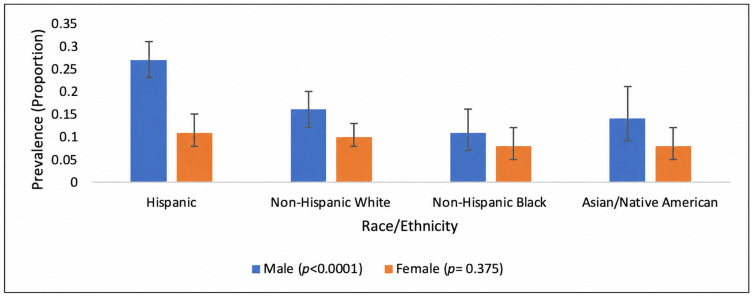
Prevalence of AFLD by Race/Ethnicity & Sex (*n* = 3967).

Stratified analyses demonstrated that Hispanic males had the highest AFLD prevalence, whereas non-Hispanic Black females had the lowest prevalence (Figure 2).

Survey-weighted regression analyses demonstrated significant associations between MOPs and AFLD prevalence. In MOP 1, compared with metabolically healthy normal-weight participants, metabolically unhealthy overweight participants and metabolically healthy obese participants demonstrated significantly higher AFLD prevalence. The strongest association was observed among metabolically unhealthy obese participants (PR: 7.03; 95% CI: 4.27–11.58).

Similarly, in MOP 2, overweight participants with MS and obese participants with MS demonstrated substantially higher AFLD prevalence compared with normal weight participants without MA. Obese participants with MS exhibited the strongest association overall (PR: 8.09; 95% CI: 5.60–11.71) (Table 4).

In adjusted models, obesity combined with metabolic dysfunction remained strongly associated with AFLD. Compared with metabolically healthy normal-weight participants, metabolically unhealthy obese individuals demonstrated approximately sixfold higher AFLD prevalence (adjusted PR: 6.00; 95% CI:3.58–10.04). Likewise, obese participants with MS had significantly elevated prevalence (adjusted PR: 6.73; 95% CI: 4.51–10.05). Overweight participants with MS also demonstrated increased AFLD prevalence, whereas normal weight participants with MS did not significantly differ from the reference group (Table 5).

Sex-stratified analyses demonstrated important differences in AFLD prevalence across MOP. In both MOP classification schemes, metabolically unhealthy obesity remained significantly associated with AFLD among both men and women; however, the magnitude of association was consistently greater among men.

MOP 2 demonstrated stronger discriminatory capacity than MOP 1, particularly among male participants. Overweight men with MS demonstrated significantly elevated AFLD prevalence, whereas this association was not statistically significant among women. Obesity combined with MS demonstrated the strongest association with AFLD in both sexes, with particularly elevated prevalence among men (Table 6).

Reclassification of our target cohort according to the updated international nomenclature revealed an overwhelmingly high metabolic burden. Out of the heavy-drinking subpopulation presenting with elevated liver enzymes (*n* = 532), the vast majority met the diagnostic criteria for some form of MetALD (97.47%; unweighted *n* = 515) due to the concurrent presence of one or more metabolic risk factors. Conversely, only a minute fraction of the disease cohort presented as pure ALD without any accompanying metabolic anomalies (2.53%; unweighted *n* = 17). Gender distribution differed significantly across severity tiers (*p* = 0.0418), with men disproportionately represented in the pure ALD and severe MetALD groups (Table 7).

## 4. Discussion

In this nationally representative study of US adults, obesity and metabolic dysfunction were strongly associated with AFLD prevalence (14%), which is higher than the global prevalence (5%). This can primarily be attributed to the study participant selection criteria which was restricted to adults aged 18–50, which is a population with higher rates of alcohol consumption than the broader adult population. Furthermore, Hispanic and non-Hispanic Black populations, who comprise a substantial proportion of the weighted US population, have been shown to bear a disproportionately higher burden of AFLD (20% and 9%, respectively), consistent with prior literature [28,29]. Participants with obesity combined with metabolic abnormalities demonstrated the greatest prevalence of AFLD, while metabolically healthy normal-weight individuals consistently exhibited the lowest prevalence. These findings suggest that metabolic dysfunction is substantially associated with alcohol-associated hepatic injury beyond alcohol consumption alone.

Our findings align with growing evidence demonstrating that metabolic dysfunction amplifies susceptibility to SLD and alcohol-related liver injury [12,30]. Obesity, insulin resistance, dyslipidemia, and hypertension are known to promote hepatic inflammation, oxidative stress, and impaired lipid metabolism, thereby exacerbating alcohol-induced hepatocellular damage [31]. The observed associations support the evolving conceptual framework of SLD, in which metabolic dysfunction plays a central role in disease progression alongside alcohol exposure.

Importantly, MOP 2, which incorporated MS criteria, demonstrated stronger associations with AFLD than MOP 1 across both sexes. One potential explanation is that ATP III MS criteria identify individuals with more advanced and clinically established cardiometabolic dysfunction. In contrast, metabolically unhealthy classifications may capture individuals with less severe or subclinical metabolic abnormalities. Consequently, MS may better identify individuals with metabolic profiles that potentiate alcohol-related hepatic injury and inflammation. This dose–response pattern was reinforced when AFLD was further stratified by the number of concurrent metabolic risk factors, which showed that over half of participants meeting AFLD criteria had three or more concurrent factors, indicating that cumulative cardiometabolic burden, not any single risk factor, distinguishes disease severity within the AFLD spectrum. This finding reinforces the rationale for the 2023 consensus nomenclature, which incorporates metabolic risk factor burden into disease classification rather than treating alcohol use and metabolic dysfunction as independent contributors [4].

Our findings are also consistent with previous epidemiologic studies reporting strong associations between obesity, dyslipidemia, diabetes, and alcohol-associated liver disease [6,32]. Furthermore, low socioeconomic status, male sex, and limited educational attainment have been associated with increased ALD burden and reduced access to preventive healthcare services [33,34].

Substantial demographic disparities were also observed. Hispanic participants, particularly Hispanic men, demonstrated the highest AFLD prevalence [28]. These findings are consistent with previous literature reporting disproportionately elevated burdens of metabolic disease and SLD among Hispanic populations [21,35]. Potential contributing factors include differences in alcohol consumption patterns, obesity prevalence, socioeconomic factors, healthcare access, and genetic susceptibility, including patatin-like phospholipase domain-containing protein 3 (PNPLA3) polymorphisms associated with hepatic fat accumulation [29,36]. Sex-specific differences were similarly notable. Men demonstrated consistently higher AFLD prevalence than women across nearly all MOPs and across the four-tier MetALD severity gradient. Biological mechanisms underlying these differences may include variations in alcohol metabolism, visceral adiposity distribution, hormonal influences, and inflammatory responses to metabolic stress [37,38].

The study has several important strengths. The use NHANES data provides nationally representative estimates and enhances generalizability to the US population. Furthermore, incorporation of MOP frameworks allowed for a more nuanced assessment of the combined influence of obesity and metabolic dysfunction on AFLD prevalence. Nevertheless, several limitations should be acknowledged. First, the cross-sectional design of NHANES precludes causal inference regarding the relationships among alcohol consumption, metabolic dysfunction, and AFLD. Second, AFLD was operationally designed using elevated ALT/AST levels combined with self-reported risky alcohol consumption rather than imaging-based liver assessment, which may have introduced misclassification bias. Although liver enzymes alone are not sufficient for definitive SLD diagnosis, NHANES lacks standardized imaging-based liver assessment across all survey cycles included in this study. Therefore, this approach was used as a pragmatic epidemiologic definition consistent with prior population-based studies. Third, alcohol consumption data were self-reported and may be subject to recall or reporting bias.

Despite these limitations, the present findings highlight the importance of integrating metabolic health assessment into alcohol-related liver disease screening and prevention strategies. Interventions targeting obesity, MS, and unhealthy lifestyle behaviors may help reduce AFLD burden, particularly among high-risk demographic groups.

## 5. Conclusions

In this nationally representative study of US adults, obesity and metabolic dysfunction were strongly associated with AFLD prevalence. Participants with obesity combined with metabolic abnormalities demonstrated the highest prevalence of AFLD, while Hispanic individuals and men represented disproportionately affected populations.

MOPs incorporating MS demonstrated stronger associations with AFLD than classifications based solely on metabolic health status, suggesting that MS may better capture clinically significant metabolic vulnerability related to alcohol-associated liver injury.

These findings support the importance of screening strategies that extend beyond alcohol consumption alone and incorporate assessment of obesity and metabolic dysfunction. Future longitudinal studies incorporating imaging-based liver assessment and contemporary SLD classifications are needed to further clarify the relationship between metabolic dysfunction and alcohol-associated liver disease progression.

## Figures and Tables

**Table 1:** Demographic summary of the participants (*n* = 3967).

	Overall (*n* = 3967)	With AFLD (*n* = 532)
	*n* (%)	*n* (%)
Age (years) [mean (SD)]	33.04 (8.34)	33.04 (8.34)
Sex		
Male	1955 (50)	339 (63)
Female	2012 (50)	193 (37)
Education		
<High school	658 (11)	124 (16)
High school graduate or GED	953 (24)	159 (30)
Some college or AA degree	1329 (33)	175 (35)
College graduate or above	1027 (32)	74 (19)
Ethnicity		
Hispanic	1076 (20)	207 (28)
Non-Hispanic White	1466 (61)	195 (58)
Non-Hispanic black	766 (10)	68 (7)
Asian/Native American	659 (9)	62 (7)
Marital status		
Never married	1232 (31)	163 (29)
Married	1482 (37.4)	189 (40)
Living with partner	533 (13.4)	95 (17)
Widow/Divorced/Separated	395 (10)	54 (9)
Unknown	325 (8.2)	31 (4)
Income		
<45K	1819 (37)	273 (40)
45–100K	1105 (31)	145 (30)
>100K	758 (26)	74 (23)
Unknown	285 (6)	40 (7)
Unhealthy behavior		
No	906 (26)	34 (10)
Yes	3061 (74)	498 (90)
Cardiac Syndrome		
No	1450 (36)	103 (18)
Yes	2517 (64)	429 (82)

**Table 2:** Distribution of Exposures in the Entire Cohort (*n* = 3967).

	*n* (%)
Obesity status	
Normal	1673 (42)
Overweight	1312 (33)
Obese	982 (24)
MS	
No	2961 (74.6)
Yes	988 (24.9)
Unknown	18 (0.5)
Metabolic Health	
Metabolic healthy	403 (10)
Metabolic unhealthy	3564 (90)
MOP 1	
NW + MH	825 (21)
NM + MU	848 (22)
OW + MH	422 (11)
OW + MU	890 (23)
OB + MH	203 (5)
OB + MU	779 (19)
MOP 2 *	
NW + No MS	1558 (39.4)
NM + MS	111 (2.8)
OW + No MS	954 (24.2)
OW + MS	352 (8.9)
OB + No MS	449 (11.4)
OB + MS	525 (13.3)

* Data were missing for these variables; therefore, the frequencies may not sum to the total sample size (*n*).

**Table 3:** Weighted prevalence of AFLD by study exposures and covariates (*n* = 3967).

Variable	Categories	Prev	95% CI	*p* Value
	Overall	0.14	0.12, 0.16	
Sex	Male	0.17	0.15, 0.20	<0.0001
	Female	0.10	0.08, 0.12	
Education	<High school	0.19	0.15, 0.24	<0.0001
	High school graduate or GED	0.18	0.15, 0.20	
	Some college or AA degree	0.15	0.12, 0.17	
	College graduate or above	0.08	0.06, 0.11	
Ethnicity	Hispanic	0.20	0.17, 0.23	0.0001
	Non-Hispanic White	0.13	0.11, 0.15	
	Non-Hispanic black	0.09	0.07, 0.13	
	Asian/Native American	0.11	0.08, 0.15	
Marital Status	Never married	0.13	0.10, 0.16	0.2192
	Married	0.13	0.11, 0.16	
	Living with partner	0.18	0.14, 0.23	
	Widow/Divorced/Separated	0.14	0.10, 0.18	
	Unknown	0.11	0.07, 0.17	
Income	<45K	0.15	0.13, 0.17	0.5666
	45–100K	0.13	0.11, 0.16	
	>100K	0.12	0.09, 0.17	
	Unknown	0.14	0.10, 0.21	
Unhealthy behavior	No	0.05	0.04, 0.07	<0.0001
	Yes	0.17	0.15, 0.19	
Obesity status	Normal	0.05	0.04, 0.07	<0.001
	Overweight	0.13	0.11, 0.16	
	Obese	0.3	0.26, 0.34	
Cardiac syndrome	No	0.07	0.05, 0.09	<0.0001
	Yes	0.18	0.16, 0.20	
(MOP 1)	NW + MH	0.05	0.03, 0.07	<0.0001
	NM + MU	0.05	0.04, 0.08	
	OW + MH	0.06	0.04, 0.09	
	OW + MU	0.17	0.13, 0.21	
	OB + MH	0.17	0.11, 0.26	
	OB + MU	0.33	0.28, 0.38	
MOP 2	NW + No MS	0.05	0.02, 0.10	<0.0001
	NM + MS	0.10	0.08, 0.13	
	OW + No MS	0.19	0.15, 0.24	
	OW + MS	0.24	0.19, 0.30	
	OB + No MS	0.14	0.12, 0.16	
	OB + MS	0.39	0.34, 0.45	

**Table 4:** Unadjusted associations of factors/covariates with AFLD (*n* = 3967).

Variable	PR	95% CI	*p*-Value
Age, mean (SD)	1.01	1.00–1.02	0.242
Sex			
Male	Reference		
Female	0.57	0.46–0.71	<0.0001
Education			
<High School	Reference		
High School graduate or GED	0.91	0.69–1.20	0.495
Some College or AA degree	0.75	0.55–1.02	0.068
College graduate or above	0.42	0.27–0.64	<0.0001
Ethnicity			
Hispanic	Reference		
Non-Hispanic White	0.66	0.52–0.84	0.001
Non-Hispanic Black	0.47	0.34–0.66	<0.0001
Asian/Native American	0.55	0.38–0.80	0.002
Marital Status			
Never Married	Reference		
Married	1.03	0.77–1.38	0.82
Living with a partner	1.37	1.01–1.85	0.044
Widowed/Divorced/Separated	1.07	0.73–1.56	0.715
Missing/Refused	0.83	0.49–1.41	0.477
Income			
<45K	Reference		
45–100K	0.90	0.71–1.13	0.35
>100K	0.82	0.58–1.16	0.254
Missing	0.96	0.63–1.46	0.84
Unhealthy Behavior	No as Reference		
Yes	3.32	2.32–4.75	<0.0001
Cardiac Syndrome	No as Reference		
Yes	2.60	1.99–3.40	<0.0001
Obesity status			
Normal	Reference		
Overweight	2.65	1.83–3.83	<0.0001
Obese	5.93	4.23–8.31	<0.0001
MOP 1			
NW + MH	Reference		
NM + MU	1.14	0.64–2.06	0.649
OW + MH	1.32	0.64–2.70	0.446
OW + MU	3.54	2.12–5.91	<0.0001
OB + MH	3.71	2.19–6.27	<0.0001
OB + MU	7.03	4.27–11.58	<0.0001
MOP 2			
NW + No MS	Reference		
NM + MS	1.00	0.45–2.23	0.997
OW + No MS	2.03	1.34–3.09	0.001
OW + MS	4.92	3.32–7.31	<0.0001
OB + No MS	3.90	2.73–5.58	<0.0001
OB + MS	8.09	5.60–1.71	<0.0001

PR: prevalence ratio.

**Table 5:** Adjusted Association of MOPs with AFLD.

Variables	PR	95% CI	*p*-Value
MOP 1			
NW + MH	Reference		
NM + MU	1.13	0.62–2.05	0.684
OW + MH	1.21	0.59–2.52	0.594
OW + MU	3.18	1.87–5.39	<0.0001
OB + MH	3.21	1.92–5.38	<0.0001
OB + MU	6.00	3.58–10.04	<0.0001
MOP 2			
NW + No MS	Reference		
NM + MS	1.05	0.46–2.38	0.913
OW + No MS	1.92	1.25–2.93	0.003
OW + MS	4.26	2.80–6.48	<0.0001
OB + No MS	3.69	2.52–5.38	<0.0001
OB + MS	6.73	4.51–10.05	<0.0001

The model was adjusted for age, sex, education, ethnicity, marital status, income, and unhealthy behavior.

**Table 6:** Adjusted Association of MOPs with AFLD Stratified by Sex.

	Male			Female		
	OR	95% CI	*p*-Value	OR	95% CI	*p*-Value
**MOP 1**						
NW + MH	Reference					
NM + MU	0.70	0.32–1.55	0.375	1.58	0.69–3.58	0.271
OW + MH	1.11	0.45–2.74	0.817	1.58	0.66–3.80	0.294
OW + MU	4.30	2.33–7.92	<0.0001	2.19	0.97–4.92	0.058
OB + MH	4.50	2.27–8.89	<0.0001	2.06	0.80–5.31	0.132
OB + MU	8.19	4.41–15.23	<0.0001	4.44	2.21–8.91	<0.001
**MOP 2**						
NW + No MS	Reference					
NM + MS	0.61	0.12–2.96	0.527	0.90	0.30–2.65	0.84
OW + No MS	2.77	1.69–4.54	<0.0001	1.42	0.76–2.66	0.264
OW + MS	7.51	4.70–11.97	<0.0001	2.15	1.12–4.11	0.022
OB + No MS	5.94	3.71–9.50	<0.0001	2.22	1.19–4.14	0.014
OB + MS	10.92	6.86–17.37	<0.0001	4.08	2.33–7.13	<0.001

**Table 7:** Baseline Characteristics Stratified by a 4-Tier 2023 Consensus Nomenclature Severity Gradient.

Baseline Characteristics	Pure ALD (0 Anomalies) *n* = 17	Mild MetALD (1 Anomaly) *n* = 84	Moderate MetALD (2 Anomalies) *n* = 158	Severe MetALD (≥3 Anomalies) *n* = 273	Total Cohort *n* = 532	*p*-Value
Cohort Fraction, *n* (%)	17 (2.53)	84 (15.79)	158 (29.70)	273 (51.32)	532 (100.00)	—
Gender, *n* (%)						0.0418
Male	12 (76.93)	58 (60.90)	86 (52.49)	183 (69.50)	339 (63.31)	
Female	5 (23.07)	26 (39.10)	72 (47.51)	90 (30.50)	193 (36.69)	
Education Level, *n* (%)						0.4599
<High School	5 (22.70)	21 (19.15)	31 (12.90)	67 (16.25)	124 (15.89)	
High School Graduate/GED	2 (23.69)	28 (28.89)	52 (36.94)	77 (26.99)	159 (30.13)	
Some College or AA degree	6 (38.83)	21 (29.28)	47 (28.77)	101 (40.42)	175 (35.18)	
College Graduate or above	4 (14.78)	14 (22.68)	28 (21.39)	28 (16.34)	74 (18.79)	
Ethnicity, *n* (%)						0.1176
Hispanic	8 (43.13)	23 (17.58)	69 (32.98)	107 (27.69)	207 (28.03)	
Non-Hispanic White	6 (47.29)	31 (67.58)	50 (51.13)	108 (59.11)	195 (57.81)	
Non-Hispanic Black	2 (7.32)	15 (8.35)	23 (9.20)	28 (5.51)	68 (7.09)	
Others	1 (2.25)	15 (6.48)	16 (6.70)	30 (7.68)	62 (7.07)	
Marital Status, *n* (%)						0.0698
Never Married	8 (53.56)	41 (40.40)	41 (25.83)	73 (25.89)	163 (28.88)	
Married	2 (14.14)	17 (32.92)	53 (36.97)	117 (45.92)	189 (40.42)	
Living with Partner	4 (19.37)	15 (12.79)	31 (21.82)	45 (16.33)	95 (17.46)	
Widow/Divorced/Separated	1 (5.55)	4 (7.50)	18 (9.23)	31 (10.18)	54 (9.36)	
Missing/Unknown	2 (7.38)	7 (6.38)	15 (6.15)	7 (1.67)	31 (3.88)	
Income Classification, *n* (%)						0.2823
<45K	10 (64.14)	43 (40.41)	74 (41.73)	146 (38.68)	273 (40.50)	
45–100K	5 (25.64)	20 (21.27)	39 (24.98)	81 (35.34)	145 (29.81)	
>100K	1 (3.91)	13 (31.87)	27 (25.43)	33 (20.01)	74 (23.08)	
Missing	1 (6.31)	8 (6.44)	18 (7.86)	13 (5.96)	40 (6.61)	
Unhealthy Behavior, *n* (%)						0.4962
No	0 (0.00)	6 (14.61)	15 (11.23)	13 (7.81)	34 (9.70)	
Yes	17 (100.00)	78 (85.39)	143 (88.77)	260 (92.19)	498 (90.30)	

Note: Percentages inside parentheses represent design-weighted, complex survey column proportions. Whole integers represent absolute unweighted sample observation counts. *p*-values reflect the Second-Order Rao–Scott adjusted design-based Pearson F-test.

## Data Availability

The raw data supporting the conclusions of this article will be made available by the authors on request.
